# A Robotic Platform for Corn Seedling Morphological Traits Characterization

**DOI:** 10.3390/s17092082

**Published:** 2017-09-12

**Authors:** Hang Lu, Lie Tang, Steven A. Whitham, Yu Mei

**Affiliations:** 1 Department of Agricultural and Biosystems Engineering, Iowa State University, 2346 Elings Hall, Ames, IA 50011, USA; neallvhang@gmail.com; 2 Department of Plant Pathology and Microbiology, Iowa State University, Ames, IA 50011, USA; swhitham@iastate.edu (S.A.W.); yumei@iastate.edu (Y.M.)

**Keywords:** plant phenotyping, corn breeding, 3D reconstruction, point cloud, robot arm, ToF camera

## Abstract

Crop breeding plays an important role in modern agriculture, improving plant performance, and increasing yield. Identifying the genes that are responsible for beneficial traits greatly facilitates plant breeding efforts for increasing crop production. However, associating genes and their functions with agronomic traits requires researchers to observe, measure, record, and analyze phenotypes of large numbers of plants, a repetitive and error-prone job if performed manually. An automated seedling phenotyping system aimed at replacing manual measurement, reducing sampling time, and increasing the allowable work time is thus highly valuable. Toward this goal, we developed an automated corn seedling phenotyping platform based on a time-of-flight of light (ToF) camera and an industrial robot arm. A ToF camera is mounted on the end effector of the robot arm. The arm positions the ToF camera at different viewpoints for acquiring 3D point cloud data. A camera-to-arm transformation matrix was calculated using a hand-eye calibration procedure and applied to transfer different viewpoints into an arm-based coordinate frame. Point cloud data filters were developed to remove the noise in the background and in the merged seedling point clouds. A 3D-to-2D projection and an *x*-axis pixel density distribution method were used to segment the stem and leaves. Finally, separated leaves were fitted with 3D curves for morphological traits characterization. This platform was tested on a sample of 60 corn plants at their early growth stages with between two to five leaves. The error ratios of the stem height and leave length measurements are 13.7% and 13.1%, respectively, demonstrating the feasibility of this robotic system for automated corn seedling phenotyping.

## 1. Introduction

Crop breeding technologies can increase yield and improve plant performance. The key to the success of crop breeding is to identify the genes that are responsible for different traits like yield of different crops. It is inefficient and error-prone to observe, measure, record, and analyze phenotypes of large numbers of plants manually, and to associate genes and their functions with agronomic traits. We proposed an automated indoor corn seedling phenotyping system that can replace manual measurement, reducing the sampling time and increasing system throughput. In this project, a time-of-flight of light (ToF) camera and an industrial robot arm are utilized to develop an automated corn seedling phenotyping platform.

Although scientists have collected abundant information of plant genotype due to the recent revolution of genomic technologies, the genomic information could not be fully capitalized upon without correct linkage between genotype and phenotype [1]. Plant breeders and ecologists have been studying plant phenotyping for many years. High-throughput phenotyping for evaluating hundreds of genotypes is routine in plant breeding [2]. 3D reconstruction has been shown to be a feasible method for plant morphological traits extraction. For example, Ijiri et al. [3] developed an application for modeling flowers in 3D. Their system provides a sketch-based interface that allows users to design and model the layouts of floral components and inflorescences, helping botanists concisely describe the structure of flowers quickly and easily. Some groups of researchers provided 3D models of rice plants from images and barley plants from 3D sensors. Watanabe et al. [4] used a 3D digitizer to measure rice plant structure to specify rice plant architecture and to find suitable functions for describing its 3D growth at all stages. Multiple-view stereo imaging was applied to reconstruct 3D models of plants from 2D images [5] and 3D plant models with high spatial resolution were also achieved [6]. However, it has proven to be challenging for stereo vision to handle the complexity of plant canopies given the difficulties in stereo matching caused by leaf occlusion and the lack of leaf surface texture. A visual hull algorithm was used to reconstruct 3D models of corn and barley plants [7], but it was found challenging to apply it to complex plant canopies [8]. Recently, plant phenotyping has gained more attention because of the development of advanced sensors and robotic data collection and plant monitoring methodologies. Robots were used in plant phenotyping systems to improve the automatic performance. Weiss et al. [9] used 3D LIDAR sensors to develop an application for reliably detecting individual plants within a row in real-time. Their results showed that this application could be assisted by using agricultural robots for localization, mapping, and navigation.

There are a variety of methods for corn plant phenotype discovery and 3D visualization. Dornbusch et al. [10] improved the modeling function of the shapes of leaves and stems of corn plants and proposed a new method for function parameterization from 3D point cloud data of the plants. Although they achieved excellent results, the images could not be automatically captured. De Morae Frasson et al. [11] also developed an application to build detailed three-dimensional digital models of corn plants by using an unmodified commercial digital camera and software. In their approach, 3D reconstruction of plants was the first step to provide morphological and position information, which was needed to accomplish other operations on plants such as imaging, probing and cutting at specific locations. Alenyà et al. [12] used ToF depth data to perform quadratic surface fitting that was applied to segment plant images [8]. They showed that the obtained surface fit well with the target leaves and the candidate leaves could be approached by a robot-mounted camera using location information. This work proved that combining dense color data and depth data could provide adequate 3D approximation to automatically complete plant measurements. Teng et al. [13] treated normalized centroid-contour distance as the classification feature for sorting different leaves in their system. Their leaf classification scheme combined 3D and color information but was not fully automated. Klose et al. [14] constructed an outdoor automatic plant phenotyping system and concluded that ToF cameras could also be useful with the help of sunlight shades in outdoor field conditions. Their system could collect data while moving at a speed of 3.6 km/h, meaning that it could be used in combination with an autonomous field robot.

This research aims to develop a fully automatic corn seedling phenotyping platform capable of generating 3D reconstructions and outputting corn seedlings’ morphological traits, including number of leaves, leaf length, and stem height.

## 2. Materials and Methods

**Figure 1:**
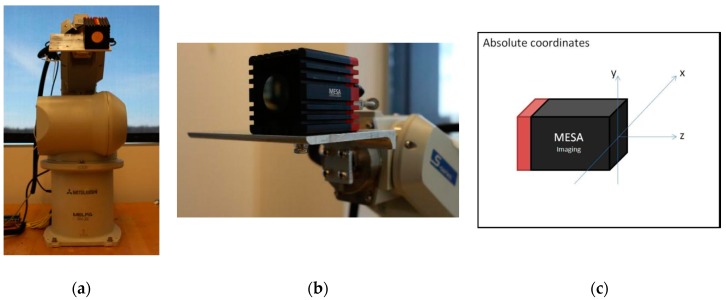
Robot arm setup (**a**); ToF camera mounted on the end effector of the robot arm (**b**); and the ToF camera coordinate system (**c**).

An overview of our platform is shown in Figure 1. The system contains a ToF camera (SR_4000, MESA Imaging, Rueschlikon, Switzerland), an industrial robot arm (RV_3SD, Mitsubishi Electric Automation, Inc., Vernon Hills, IL, USA), and a computer station. To start the process, the user initiates a request to the robotic system. Then the system sends commands to the robot arm, including commands to specific positions with various poses to acquire a 3D point cloud from these viewpoints. The point clouds are then transformed and merged into arm base coordinates. The platform performs filtering, stem and leaf segmentation, phenotypic data extraction, and visualization.

### 2.1. Plant Material and Data Collection Schedule

A total of 60 sweet corn (*Zea mays* L. “Golden × Bantam”) seedlings were used in this study. These corn plants were grown in growth chambers set to a constant temperature of 22 °C and with a 16 h photo period. Image and hand measurement data were collected starting at seven days after germination. Every three days we measured the stem height and leaf length on each plant using the developed robotic phenotyping system and also by hand. The measurements collected by hand were treated as the reference values. There were nine measurements taken from the plants on days 8, 11, 14, 17, 20, 23, 26, 29, and 32. In total, 534 stem height and 1969 leaf measurements were taken over the course of the experiment (two plants died on day 29, and then two more died on day 32).

### 2.2. Data Collection Station Setup

Figure 1 shows how the ToF camera connects to the end effector of the robot arm. The aluminum mounting bracket has a 90 degree “L” shape with 5-inch width and 0.25-inch depth. It was designed and machined by using a manual mill (Clausing Industry Inc., Kalamazoo, MI, USA), with four holes on one side connecting to the robot arm end effector, and three holes on the other side for fixing the ToF camera.

Table 1 lists the technical specifications and some key performance indicators (tested at 25 °C) of the SR_4000 camera.

### 2.3. Hand–Eye Transformation

To estimate the 3D position and orientation of the target object relative to the robot base coordinate frame, it is essential to know the relationship between the robot end effector and the robot base, the relationship between the camera and the robot end effector, and also the relationship between the target object and the camera. The transformation matrix between the robot end effector and the base frame can be obtained from the robot controller output without any programming or computing. The main function of the ToF camera is to output the 3D point clouds that provide the position and orientation of the target object in the camera coordinate frame. Thus, the transformation matrix of the camera and the robot end effector must be measured or calibrated to transfer target object position and orientation information to the robot base frame.

#### 2.3.1. Dimension Method

According to the dimensions and coordinate definition of the ToF camera in the manual, the origin is the center of the surface of the lens cover and *xyz* directions are shown in Figure 1. The transformation matrices from the camera’s *xyz* coordinates to the robot arm’s end effector includes both rotational and translational matrices (mm as unit) which were calculated based on manual measurements of

$$R_{D}=\left[\begin{array}{l} -0.9998,0.0174,-0.0006\\ -0.0175,-0.9992,0.0349\\ 0.0000,0.0349,0.9994 \end{array}\right]$$

$$T_{D}=\left[\begin{array}{l} -30.69\\ -69.03\\ 120.96 \end{array}\right]$$

#### 2.3.2. Fully Vision-Based Calibration

**Figure 2:**
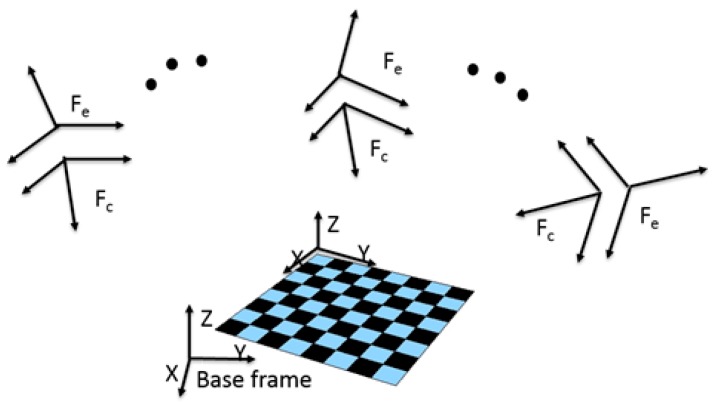
Hand–eye calibration to obtain the relationship between camera coordinate system (F_c_) and robot arm end-effector coordinate system (F_e_).

Through a standard camera calibration procedure, there are two main outputs, i.e., intrinsic and extrinsic parameters. The intrinsic parameters are used to calibrate lens distortion, while the extrinsic parameter can be used to associate the camera position with 3D world space. The camera’s intrinsic parameters are calibrated by the manufacturer. Our vision-based robot hand-eye calibration involved a standard camera calibration procedure to obtain the extrinsic parameters and incorporation of a constant relationship between camera frame and robot end effector frame (Figure 2). We used a hand–eye calibration toolbox [11] to calculate the camera to robot end effector transformation matrix. Here F_e_ is the robot arm end-effector coordinate system, and F_c_ is the camera coordinate system.

This toolbox can solve eight sets of homogeneous transformation equations: AX = XB, where X is the target matrix. The final result of the camera related to robot end effector transformation matrix had an error of less than 20 mm in each direction and we assumed the ground truth is the manual measurement.

**Figure 3:**
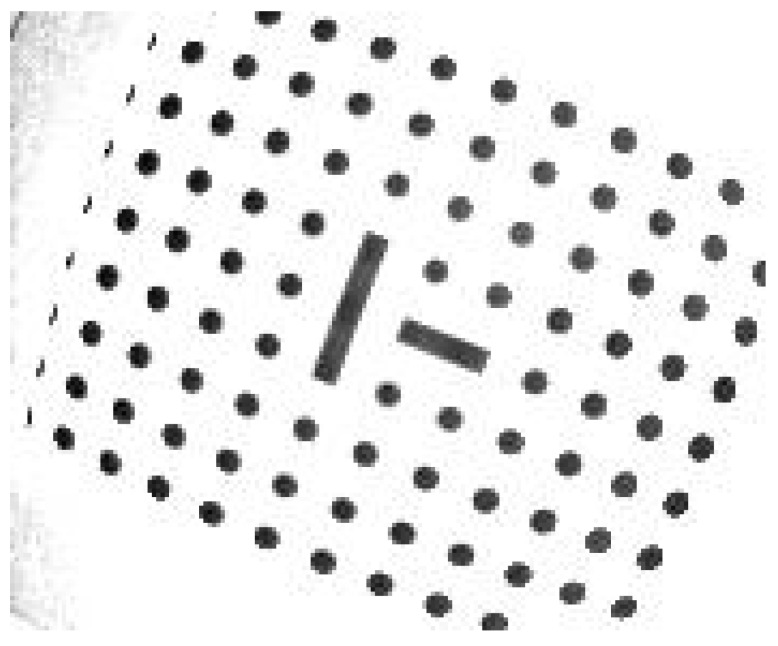
ToF camera amplitude image of a checkerboard with a low resolution.

The toolbox uses the reference pattern method to locate the location of the origin and the directions of the coordinates. In the toolbox, the pattern is a map of black dots with a cross in the center. We input the black dot diameter and distance between two dots as parameters into the toolbox. Then it detects each dot from multiple images and calculates intrinsic and extrinsic parameters of the camera. The toolbox can compute the transformation matrix between the camera and pattern map coordinates by applying extrinsic parameters. Also, we input the robot arm positions into the toolbox. Finally, it solves equation AX = XB to obtain the transformation matrix of the end effector and the camera. However, this method becomes less effective when dealing with cameras with lower resolution, because the algorithm relies on the accuracy of the detection of metric features such as corners and circle centers. Larger error is generated when detecting and locating those features in a low-resolution image acquired by a ToF camera such as SR_4000, which has a resolution of only 176 × 144 pixels (Figure 3).

Comparing these two calibration methods, the error of the dimension method is apparently smaller than that of the vision-based ToF camera. Kahn reported that there was an approximately 10 mm error of SR_4000 2D and 3D image-based hand-eye calibration in their experiment [15]. The dimension method was therefore applied in this project.

### 2.4. System Architecture

**Figure 4:**
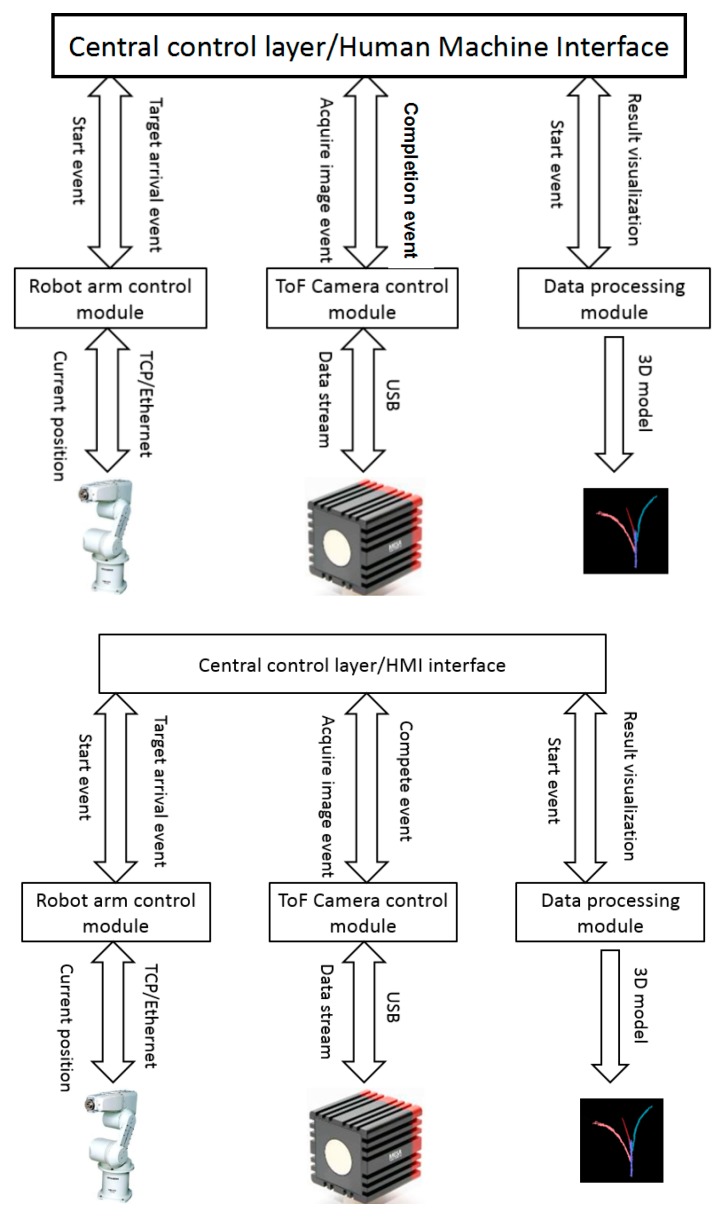
System overview.

This system contains four hierarchical modules: a main control module and user interface, a robot arm control module, a ToF camera control module, and a data processing module (Figure 4). The entire system was constructed by using multiple threads in which the central control module was the main thread and submodules were the child threads. The software was developed in the Qt development environment (www.qt.io/ide/) with programming language C++.

#### 2.4.1. Main Control Layer

This main module and interface is the central controller responsible for communicating with the robot arm, decision-making for ToF camera actions, and triggering data processing and result visualization.

During the operation, the main control layer sends a request to the robot arm control module that will then produce a program for jogging the arm to specific positions. Meanwhile, the robot arm sends its current position to the main control module in real time. A judgment is made in the main control layer on whether the arm properly determines the target sampling position. As soon as the arm determines the target locations, the main control module issues a request instruction to the ToF camera control module to acquire a 3D image. When the 3D images of multiple views are ready, the processing module begins data filtering, leaf and stem segmentation, and parameter computation. Finally, the phenotypic data are displayed in a table while the 3D reconstruction model is shown in a visualization window in the user interface.

The HMI (human–machine interaction) interface contains robot arm controller IP and port setting, command buttons, camera status-checking, a phenotype parameter output table, and 3D model visualization.

#### 2.4.2. Robot Arm Control Module

The Mitsubishi RV3S is an industrial vertical six-joint robot arm with a maximum speed of 5.5 m/s and 0.02 mm position repeatability. The programming platform, RT ToolBox2 is an independent software package with its own uniform robot programming language. It is impossible to use the RT-toolbox in our platform because our system requires real-time communication, online decision-making, and path planning. We therefore applied robot protocol and send protocol commands to the robot controller through a TCP socket. The advantage of coding with the robot protocol is that programmers can embed the robot control commands in their customized software using a programing language of their choice.

**Figure 5:**
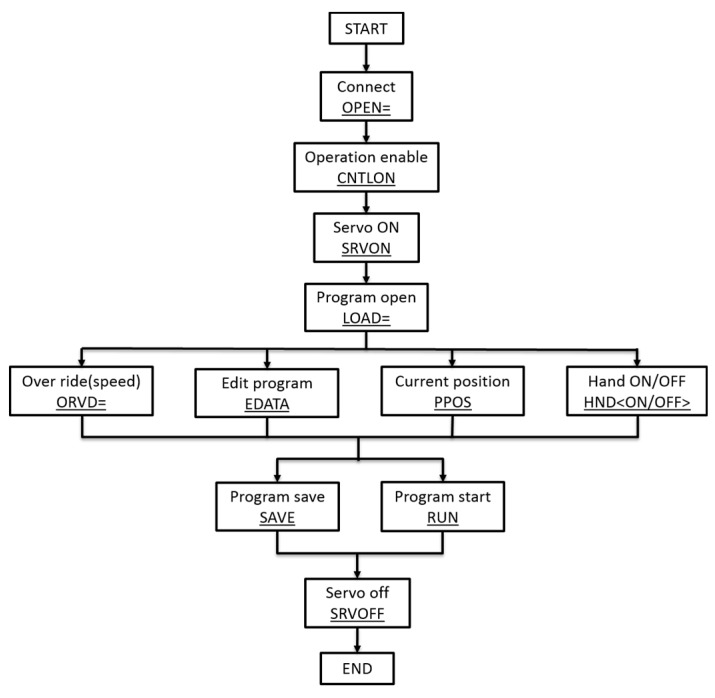
Robot arm programming flow chart.

The robot arm controller worked as a TCP server and the robot control module worked as a TCP client; they were connected by Ethernet cable. In the communication mechanism, the client must open a channel first followed by operation enable, turning on servo, movement programming, turning off servo and disconnection. Figure 5 shows the robot arm protocol programming structure.

#### 2.4.3. ToF Camera Control Module

The camera control module constantly provides a status signal to the main control module after the system turns on. When these two conditions are satisfied: camera status is set to “succeed” and when the robot arm has arrived at the target position, the central module generates an event and the camera module triggers the sensor to acquire an image in response to this event. A calibrated output stream will be transmitted to the camera module from the sensor through a USB connection, then the camera module will send an event to the main module after completing image acquisition.

When the main module responds to the imaging completion event, the data processing module becomes active; the details of the processing algorithm are discussed in the next section. Processed results will be transmitted to the main module for visualization in the user interface.

#### 2.4.4. Data Processing Module

##### 3D Image Pre-Processing and Segmentation

Point cloud data pre-processing and leaf and stem segmentation is one component of the data processing module. Pre-processing provides background and noise filtering as well as multiple-views data merging. In the leaf and stem segmentation algorithm, the 3D point cloud data were first projected into a 2D *z*-*y* plane, then a *y*-axis pixels density distribution based method was developed to obtain stem positions on the *y*-axis. After isolating the stem point clouds, the remaining points will be separated into leaf clusters.

##### Multi-View Images

**Figure 6:**
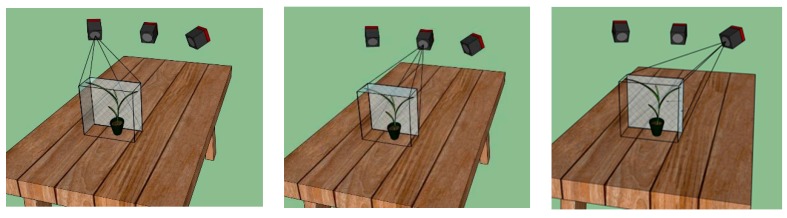
3D point cloud data acquisition from three viewpoints.

To reconstruct more details, multi-view images instead of a single front view were produced. The corn seedling is placed about 550 mm to 800 mm in front of the origin of the robot arm base coordinate system. The movement range of the robot arm is 0–859 mm along the *z* axis, −642 to 642 mm along the *y* axis, and −330 to 642 mm along the *x* axis. In fact, the movement is more limited when all six joints work together and when considering the rotations, i.e., yaw, pitch, and roll of the end effector frame. In this project the system acquired the 3D plant data from three viewpoints with a corn plant placed in front of the robot arm. Figure 6 shows the right, middle, and left views of the plant in the robot arm base coordinate system.

At each viewpoint, the robot arm produces a homogenous transformation matrix to describe the relationship between the end effector frame and the robot arm base coordinates. All data from different viewpoints can be transformed into the base coordinate system by implementing Equation (1), where ${\left[\begin{array}{c} X\\ Y\\ Z \end{array}\right]}_{base}$ represents the robot base coordinate system; ${\left[\begin{array}{c} x\\ y\\ z \end{array}\right]}_{camera}$ represents the camera coordinate system; ${\left[T\right]}_{cam-to-end}$ is the transformation matrix between the camera coordinate system and the end-effector coordinate system; and ${\left[T\right]}_{end-to-base}$ is the transformation matrix between the end-effector coordinate system and the robot base coordinate system.

(1) $${\left[\begin{array}{l} X\\ Y\\ Z\\ 1 \end{array}\right]}_{base}={\left[T\right]}_{cam-to-end}\times {\left[T\right]}_{end-to-base}\times {\left[\begin{array}{l} x\\ y\\ z\\ 1 \end{array}\right]}_{camera}$$

The camera to end-effector transformation matrix is calculated by a calibration method described in Section 2.3 and the end effector to base transformation matrix is produced by the robot arm controller.

##### Background and Noise Removal

The working area is a rectangle (250 mm × 150 mm) in front of the robot base. The maximum size of a corn plant was limited to 600 mm × 500 mm. In this way, only the point clouds that were inside the cuboid of 250 mm (width) × 500 mm (length) × 600 mm (height) were retained.

It is typical for a ToF camera to generate point clouds with varying densities; the raw data of the corn plant point cloud always contains a few sparsely-distributed outlier points. The statistical outlier removal algorithm treats a point as an outlier or inlier according to the distance to its k-nearest neighbors [13]. This threshold is set as μ + βσ, where μ is the average and the σ is the standard deviation of the k neighbor distances. The k-nearest neighbors distance value of sparse points is normally greater than the threshold, μ + βσ.

The value of β can greatly affect the results of the filter. If the β is too small, only a few noise points are removed; if too high, some points of the plant could be mistakenly removed. We used k = 10 and β = 10 as the filter parameters, as recommended by Chaivivatrakul et al. [16].

##### Leaf and Stem Segmentation

Parameter computation and trait extraction are based on segmentation, because an individual component, such as a leaf, is needed to extract the morphological features of a plant. In 3D leaf and stem segmentation, Chaivivatrakul et al. [16] sliced the corn point cloud from the bottom of the stem to the top of the leaf, and performed least-squares ellipse fitting for each module. The linked ellipses with close centers and similar semi-major axis lengths were considered as stem parts. Li projected the 3D corn point cloud into six binary images from 0, 60, 120, 180, 240, and 300 degrees of view angles [17]. If a straight line had over 50 pixels and with an inclination angle between −5° and +5°, the system would treat this line as the stem [17].

**Figure 7:**
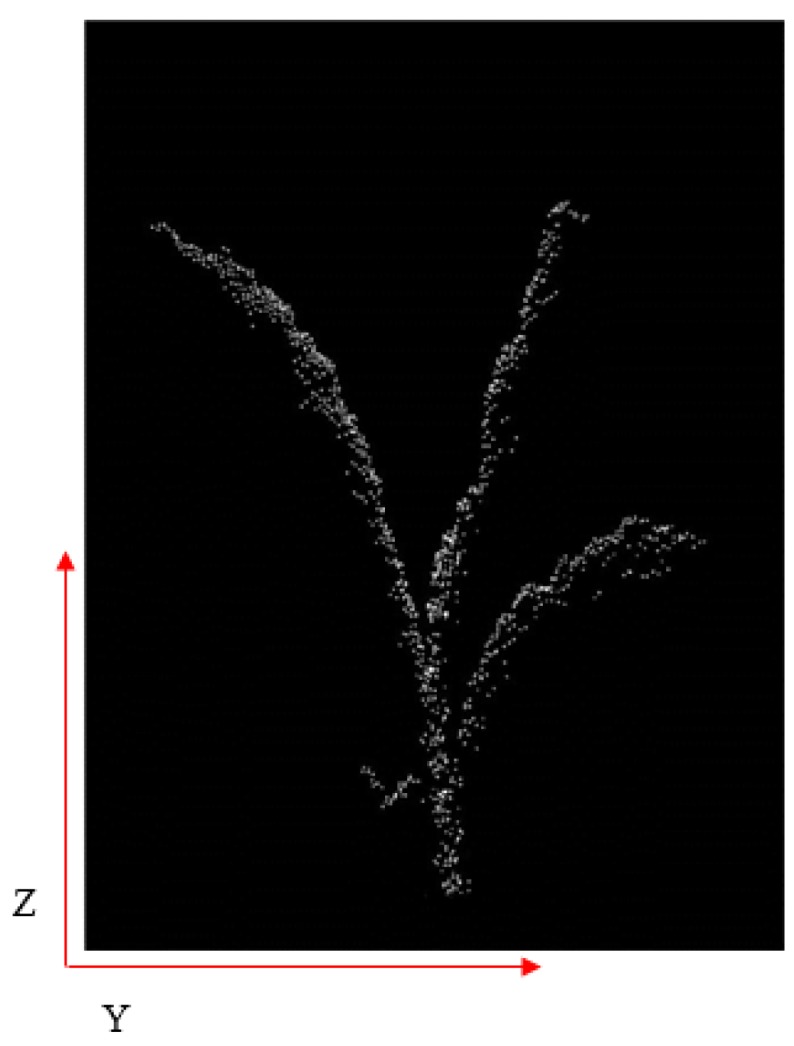
2D projection binary image.

In our segmentation algorithm, the corn plant point cloud was projected as a binary image. The white pixels belonging to the plant were given a value of 1 and the black pixels were given a value of 0 in the image. In this research, the stems of our corn plants normally stood with an angle between 85°−95° with respect to ground (Figure 7).

**Figure 8:**
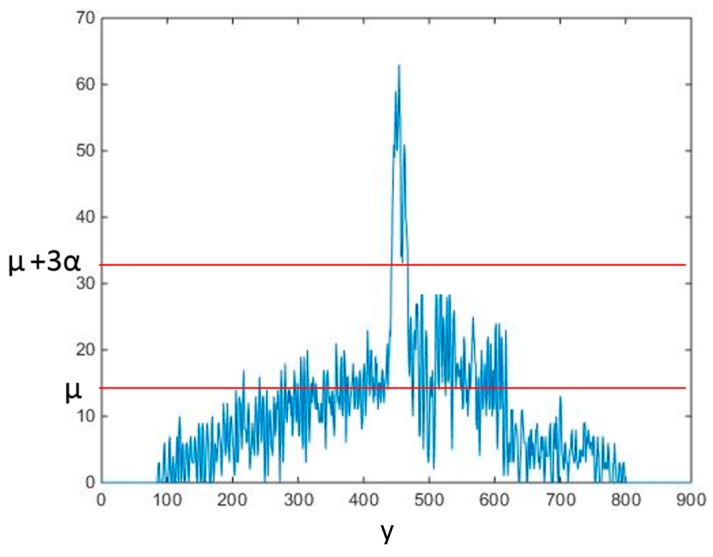
Projected point distribution in y direction.

We next calculated how many white pixels were in each unit in the y direction, generating a pixel density distribution map along the *y* axis. Because the stem part was approximately vertical, it must have the highest density in the distribution map. The *y* value of the highest density area was the location of the stem on the *y* axis (Figure 8).

**Figure 9:**
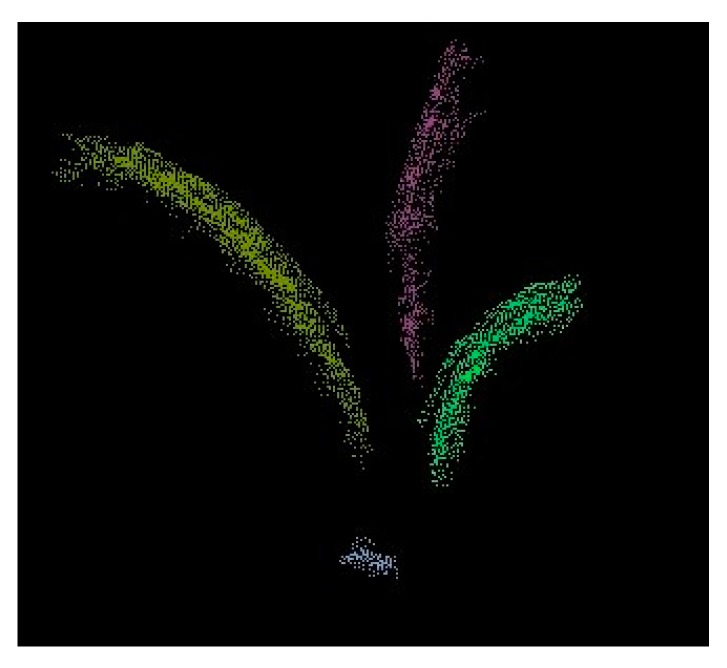
Separated leaf clusters.

After extracting the stem point cloud, the remaining points correspond to the leaves (Figure 9). To separate the leaf points into several single ones, a clustering method called “Euclidean Cluster Extraction” was implemented in this algorithm [18]. The algorithm basically defines how a point belongs to a particular point cluster and why it is different from other point clusters. Let p_i_ be a point in the point cloud Ƥ (p_j_ ∈ Ƥ), if the minimum distance from a set of points {p_i_} to p_j_ is larger than the threshold d^th^, p_j_ must belong to another cluster_._

We created a Kd-tree T to represent the input leaves point cloud Ƥ and built an empty list of clusters L to store the output. If for a point p_i_ ∈ Ƥ, we added it to a queue Q and searched for the set p_k_ that was the neighbors of p_i_ in a sphere with radius less than d^th^. When that step was completed, we added the Q to the leaf cluster L_k_. After traversing all p_i_, the segmented leaf clusters were stored in L.

##### Leaf Curve Fitting and Parameter Computation

**Figure 10:**
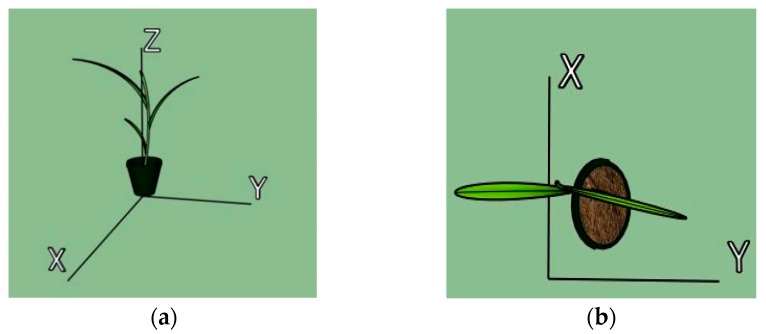
plant projected in *y*–*z* plane (**a**) and *x*–*y* plane (**b**).

Before extracting leaf parameters, the algorithm used a high-order 3D curve to describe the skeleton of each leaf. In the *x*–*y* plane, the skeleton of the leaf is a line, and if the leaf is viewed in the *y*–*z* plane, the leaf skeleton is a curve (Figure 10). We can thus split the high-order 3D curve into two equations

x = ay + b(2)

z = c_0_ + c_1_y + c_2_y^2^+ … +c_k_y^k^ (3)

**Figure 11:**
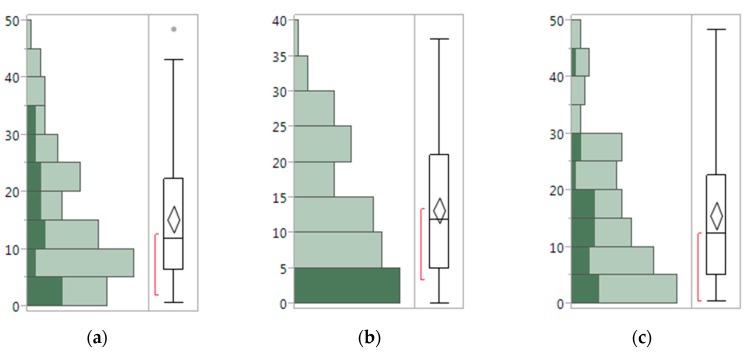
Leaf length error (%) distribution with different order of curve fitting: (**a)** second order, (**b**) third order, and (**c**) fourth order.

In the *y*–*z* plane, the leaf skeleton with greater curvature must have a larger k value, where k is the order of the curve in the *y*–*z* plane. There were 97 leaves, ranging in length from 50 mm to 521 mm, chosen randomly to test which order of curve fitting is more suitable in this project. The error was calculated by comparing the algorithm output with the manual measurements (Equation (4)); and the error distribution plot (Figure 11) and a summary statistics table (Table 2) are given below.

(4) $$Error=\frac{System\;output-manual\;measurement}{manual\;measurement}\times 100\%$$

The mean of the third curve fitting error is 13.2%, which is smaller than that of the second order (15.3%) and fourth order (15.6%) curve fitting, thus third order curve fitting was adopted in this project. The boxplot shows the error distribution. When the second order fitting was applied, the error focuses on 5% to 10%, whereas when the third and fourth order fitting were applied, the error concentrates more on 0 to 5%. This means the third and fourth order curve fitting bring smaller errors than that of the second order fitting.

**Figure 12:**
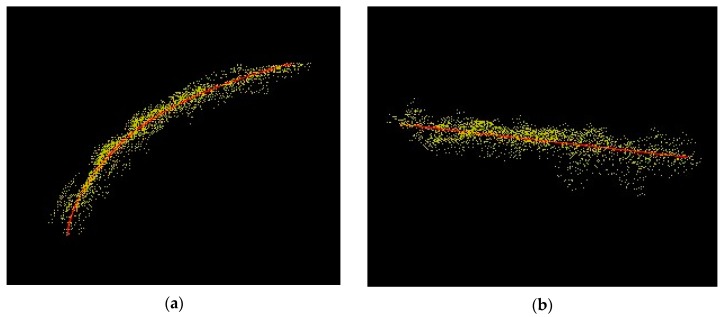
Red line is the leaf fitting in *y*–*z* plane (**a**) and *x*–*y* plane (**b**).

The *y* range of the leaf was divided into N subsections. For each *y* value, there were corresponding *x* and *z* values to make up a leaf point (*x*, *y*, *z*). When these points were connected, they formed the 3D leaf skeletons (Figure 12).

Based on the leaf skeleton fitting curve, the length of the leaf is the sum of *N* fractional lengths.

(5) $$Length=\overset{N}{\underset{i=1}{\sum }}\sqrt{{\left(x_{i}-x_{i-1}\right)}^{2}+{\left(y_{i}-y_{i-1}\right)}^{2}+{\left(z_{i}-z_{i-1}\right)}^{2}}$$

**Figure 13:**
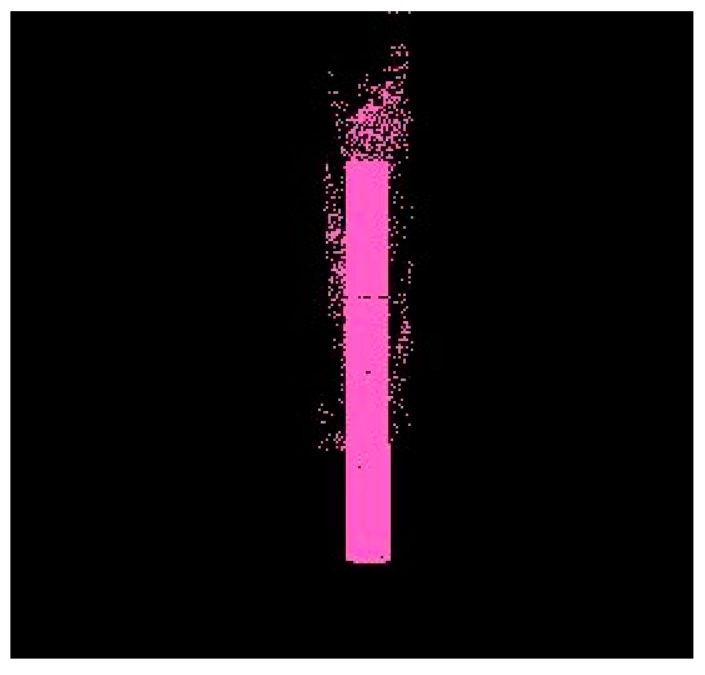
Stem fitting model.

For stem model estimation, we fitted it as a cylinder and compensated the bottom part through a lost filtering (Figure 13). The length of the stem is the highest *z* value minus the distance between the desktop and the bottom of the stem.

## 3. Results and Discussion

**Figure 14:**
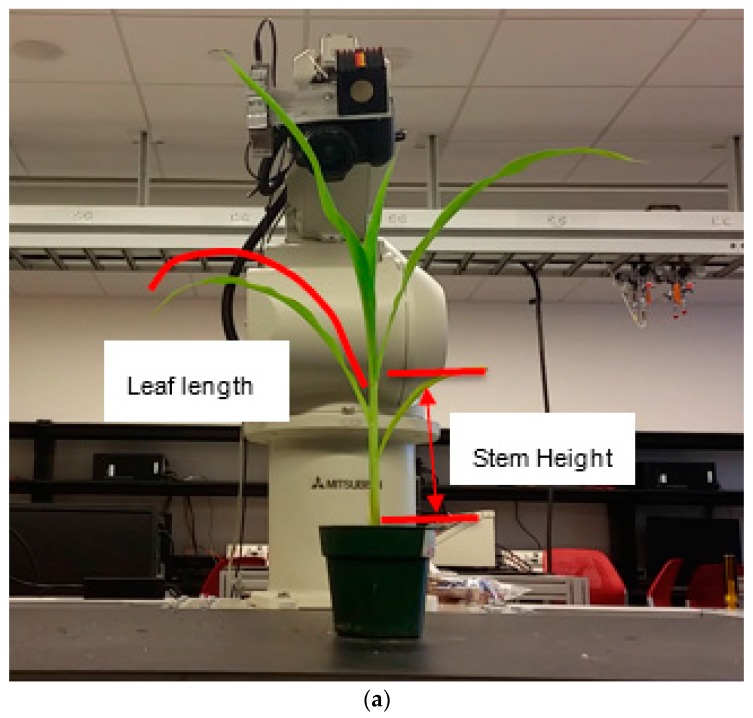

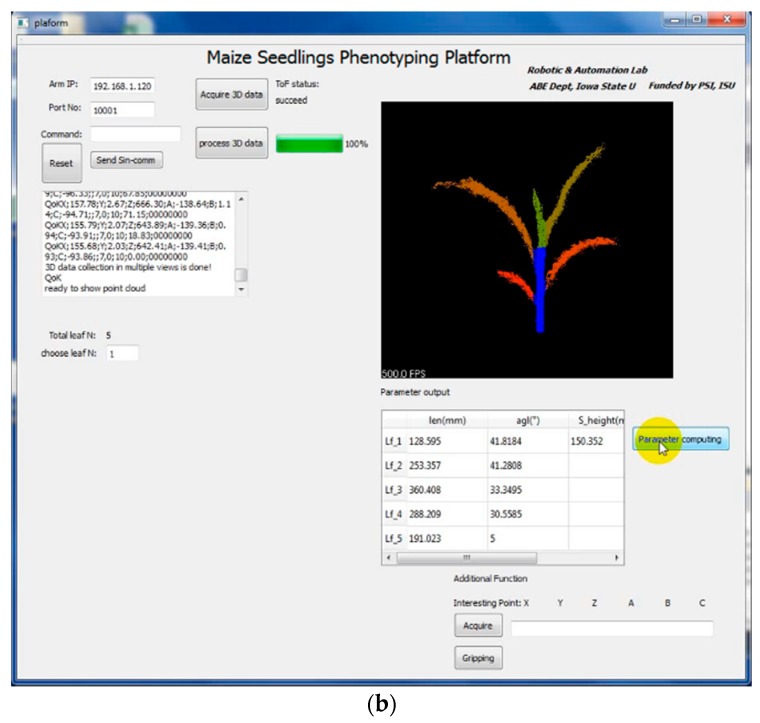
Robotic scanning station (**a**) and the software interface (**b**).

The robot arm automatically brought the ToF camera to different positions to begin collecting the 3D point cloud data of each plant. Then the software would reconstruct the 3D model of the plant by using the point cloud data from multiple views. Figure 14 shows the developed hardware and software components with an illustration where leaf length and stem height are defined. The stem and different leaves were labeled with different colors. At the same time, a table displayed the parameters of the plant, e.g., the length of each leaf and the stem height (Figure 14).

**Figure 15:**
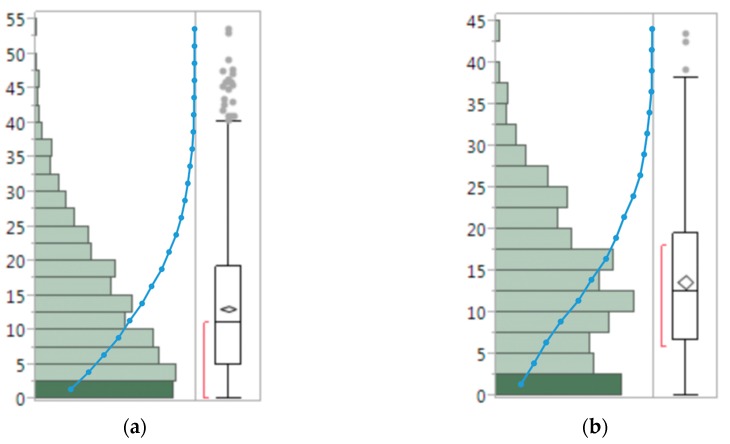
Error (%) distribution of the stem height (**a**) and leaf length (**b**) measurements generated by the system. The blue dotted lines are cumulative error distribution curves.

Figure 15 is the error distribution of stem height and leaf length. These are half-normal distributions and their mean and median values are close. There is no obvious bias shown in the error distributions, meaning that the measurements did not overestimate or underestimate the true values. When comparing the cumulative error distribution curves of stem height and leaf length measurements, the stem height measurement error ratio has a substantially higher ramp rate in the first quartile, indicating a larger concentration of stem height measurement than that of leaf length measurement in the low error range.

The stem heights of the corn plants at the time of measurement ranged from 30 mm to 220 mm, the length of the leaves at the time of measurement ranged from 20 mm to 567 mm. Table 3 shows that the error of stem height measurement by the system is 12.5% (median) and 13.7% (mean). The minimum error between the system outputs and the manual measurements were approximately 0%. More than 75% of stem height measurements exhibited error of less than 20%. A quarter of the stem height measurements have very small errors (6.6%). The confidence interval (95%) of the error mean is 12.9–14.4%. The leaf length measurement error is 11.1% (median value) and 13.1% (mean value) (Table 4). A quarter of the measurements of leaf length have an error of 5%, and three quarters of the measurements have an error rate less than 20%. However, there are outliers with error values of over 40% in the error distribution. Such large errors usually happened when the stems were as short as 30–60 mm. The ToF sensor contributes 10 mm error, close to the stem height, causing a large relative error value when stems are short. The error comes from the ToF camera (10 mm), filtering and leaf curve fitting. The choice of filter parameters and the order of fitting curve to satisfy all situations is difficult because each plant has differently shaped leaves. The surface of a corn leaf is not flat, and the fluctuating part of the leaf may require higher orders of curve fitting.

## 4. Conclusions

In this project, we were able to use an automated system to generate phenotypic data of stem height and leaf length of corn seedlings. When comparing the values acquired by the developed robotic measurement system and those obtained by manual measurement, the robotic system performed satisfactorily, proving its utility in automated plant phenotyping for corn plant seedlings.

The larger outlier errors were likely caused by the filters, the accuracy of ToF camera, and the curve fitting algorithm. The ToF camera we used in this project has a resolution of only 176 × 144, so a higher resolution 3D sensor will improve the accuracy of this robotic measurement system. The filter parameters are also important to the measurement output of the system. We used pass-through and outlier removal filters in this project and, because these two filters are sensitive to plant shape, it is difficult to fix the parameter values of the filter while satisfying all situations. If color data could be used to remove the noise, the measurement error would decrease. This could be accomplished by using a RGB threshold to retain only point cloud points that are of green color (corn plants) and remove background and other noise. The current system considers only the shape of the plant but, in addition to measuring plant structural features, there are some other traits that can be of interest to plant scientists—including responses to pathogens, pests, and environment stresses—that can be manifested as changes in color. Different sensors, such as hyperspectral cameras, can be added to the system to observe changes in color, chemical composition, and photosynthetic activities.

The developed phenotyping platform requires that plants are brought to a staging area next to the robot arm, which is fixed to a work table. However, for phenotyping applications, it will be helpful if this system becomes mobile. A mobile system can collect data on plants in growth chamber, greenhouse, or field without the need to move plants. The mobile application will require coordination of the robot arm, the end effector, and the mobile rover to repeatedly visit plants for data collection over the course of the plant growth period.

## Figures and Tables

**Table 1:** SR_4000 data sheet.

Illumination Wavelength	850 nm
modulation frequency	30 MHz
detection range	0.1–5.0 m
calibrated range	0.8–5.0 m
absolute distance measurement accuracy	±10 mm with less than 0.5 mm/°C drift with temperature
field of view	43° (h) × 34°
maximum frame rate	50 FPS
pixel array size	176 (h) × 144 (v)
operating temperature	+10 °C to +50 °C

**Table 2:** Leaf length error estimated by different orders fitting (%).

**Quantiles**									
**k = 2 (Second Order)**			**k = 3 (Third Order)**			**k = 4 (Fourth Order)**		
100%	Maximum	48.4	100%	Maximum	37.4	100%	Maximum	48.4
75%	Quartile	22.4	75%	Quartile	21.1	75%	Quartile	22.7
50%	Median	11.9	50%	Median	11.8	50%	Median	12.4
25%	Quartile	6.4	25%	Quartile	5.0	25%	Quartile	5.1
0%	Minimum	0.6	0%	Minimum	0.0	0%	Minimum	0.4
			**Summary Statistics**					
Mean		15.3	Mean		13.2	Mean		15.6
Std. Deviation		11.5	Std. Deviation		9.1	Std. Deviation		12.1
Std. Err Mean		1.2	Std. Err Mean		0.9	Std. Err Mean		1.2
Upper 95% Mean		17.6	Upper 95% Mean		15.0	Upper 95% Mean		18.0
Lower 95% Mean		12.9	Lower 95% Mean		11.3	Lower 95% Mean		13.2
N		97	N		97	N		97

**Table 3:** Stem height error table (%).

Quantiles			Summary Statistics	
100%	Maximum	43.6	Mean	13.7
75%	Quartile	19.5	Std Deviation	8.9
50%	Median	12.5	Std Err Mean	0.4
25%	Quartile	6.6	Upper 95% Mean	14.4
0%	Minimum	0.02	Lower 95% Mean	12.9
			N	534

**Table 4:** Leaf length error table (%).

Quantiles			Summary Statistics	
100%	Maximum	53.5	Mean	13.1
75%	Quartile	19.2	Std Deviation	9.9
50%	Median	11.1	Std Err Mean	0.2
25%	Quartile	5.0	Upper 95% Mean	13.5
0%	Minimum	0	Lower 95% Mean	12.7
			N	1969
